# Clinicopathological and Molecular Insights into Gallbladder Cancer

**DOI:** 10.3390/cancers15102728

**Published:** 2023-05-12

**Authors:** Philip R. de Reuver, Rachel S. van der Post

**Affiliations:** 1 Department of Surgery, Radboud University Medical Centre, 6500 HB Nijmegen, The Netherlands; 2 Department of Pathology, Radboud University Medical Centre, 6500 HB Nijmegen, The Netherlands

Although gallbladder cancer (GBC) is rare, it is one of the few cancers with a higher mortality rate than incidence, accounting for 1.2% of all cancers and 1.7% of all cancer mortality, respectively [1]. The incidence of GBC shows remarkable geographic variation [2]. Differences in genetics, lifestyles, and environmental factors all likely contribute to this remarkable range in incidence rates. While GBC is treated by radical surgical resection, of which the extent is primarily determined by tumour stage, systemic therapy has recently received increasing attention as a method to complement surgery and increase local and distant control. In recent years, clinicopathological and molecular insights into GBC increase the capacity to enable mutation-matched personalized therapy in the future.

This Special Issue addresses various aspects concerning current state of the art and future perspectives in gallbladder cancer’s clinical and translational research. This Special Issue reports on the treatment of resectable GBC, paradigms in systemic therapy of GBC, and therapeutical implications of new insights into genetic alterations of this rare cancer.

First, de Bitter et al. illustrated the value of appropriately reporting pathological findings in GBC [3]. Their review of 849 mostly nonstructured histopathology reports of GBC resections showed that key information on prognostic factors, such as tumour side (hepatic vs. serosal), the status of the cystic duct and liver margins, and venous and perineural invasion, were frequently lacking. An accurate description of resection specimen is of importance, especially in incidentally diagnosed GBC patients (15–20% of all GBC diagnoses) who undergo a routine cholecystectomy for presumed benign gallbladder disease. The pathology report is the starting point for predicting survival after resection and may help to decide on further management, such as re-resection, lymphadenectomy, and adjuvant therapy [4]. Bitter et al. illustrated the need for standardized pathology reporting of GBC, which has already proved to lead to complete and accurate reports and improved survival in other tumour types [5].

Incidental GBC is the most common presentation of resectable gallbladder cancer. Pathogenesis, surgical and systemic treatment, and prognosis are addressed in a review by Vega and colleagues [6]. Nicely illustrated, the authors reported on the prognostic factors for a tumour at the index cholecystectomy, minimally invasive extended resections, and the role of robotic surgery. The authors mainly concluded that laparoscopic (extended) resections for selected patients with incidental GBC are safe and oncologically effective in centres with expertise. They noted that resection alone is unlikely sufficient for patients with residual disease or stage III–IV disease. This conclusion is in line with several initiatives aiming to investigate the survival benefit of neo-adjuvant treatment strategies in GBC [7].

Kuipers et al. explored the possibilities for novel treatment options in GBC by systematically analyzing current literature on molecular alterations in GBC, emphasizing targetable alterations [8]. They observed that although GBC is molecularly extremely heterogeneous, a significant proportion of tumours carry molecular alterations for which therapies are available in other tumour types. Most studies have been of relatively small sample size and derived from high-incidence populations with a disease aetiology that differs from low-incidence populations. To further expand the knowledge on the molecular landscape of a low-incidence GBC population, de Bitter et al. studied molecular characteristics in a nationwide cohort of GBC patients [9]. Indeed, their results support the findings from the literature that GBC is molecularly highly heterogeneous. Strikingly, half of the patients carried at least one molecular alteration that is targetable in other tumour types, underscoring the need for molecular testing in daily practice to expand experimental treatment possibilities for this rare and lethal cancer type.

Additionally, the reviews by Sturm et al. and Hu and Lim highlight important challenges of systemic GBC treatment options [10,11]. Targeting the HER2 pathway is a leading strategy for GBC, for which several clinical trials are ongoing to study the best combination therapy regimen. Immunotherapy regimens have been studied, but the biggest challenge is to find a promising predictive biomarker other than PD-L1 expression to identify target patient groups that benefit from checkpoint immunotherapy. Another important point that the review by Hu and Lim raises is patients’ ethnicity as a biological variable that could impact the natural course and treatment response, as discerned from the TOPAZ-1 study [12]. The molecular underpinnings of this difference still need to be better understood and require intensive research to aid patient selection in future clinical trials.

The role of endoscopy and currently available techniques in the context of palliative care of advanced gallbladder malignancy were reviewed by Schepis et al. [13]. The authors discussed essential recommendations when choosing available EUS procedures, depending on local facilities. This paper could guide clinicians when treating jaundice and cholangitis in GBC patients and when choosing endoscopic possibilities if a patient is presented with gastric outlet obstruction or incoercible pain. As more than 50% of GBC patients are diagnosed with advanced disease, innovation in minimally invasive palliative treatment is mandatory.

This Special Issue touches on relevant clinical dilemmas in treating patients with GBC. It shows the three essential steps to improve the care of patients with this highly lethal disease. A synoptic pathology report will better reveal tumour characteristics and assist in making a formal diagnosis of (incidental) GBC. Second, GBC care for individual patients should be centralised in specialised expert centres. As a substantial number of GBC patients will initially be diagnosed in non-academic peripheral hospitals, a multidisciplinary tumour board can facilitate shared clinical decision-making for GBC, including adjuvant treatment options after cholecystectomy of incidentally diagnosed GBC. Clinical findings and imaging and pathology findings can be discussed during a multidisciplinary team meeting. The team can also decide to follow up in the case of early incidentally discovered tumours (i.e., pathological stage pT1), advise a radical re-resection in the case of residual disease, or discuss systemic therapy options. Finally, a diagnosis of GBC means an extremely worrisome prospect. Our group and others have shown that although GBC is molecularly highly heterogeneous, numerous clues for molecular targeted therapies are present that should be explored by including GBC patients in multicentre trials. Therefore, every patient with GBC should undergo a molecular tumour test to enable genome-informed treatment selection and prognosticate advance outcomes of personalized systemic treatment.
